# Effects of Oil Droplet Size and Interfacial Protein Film on the Properties of Fish Myofibrillar Protein–Oil Composite Gels

**DOI:** 10.3390/molecules25020289

**Published:** 2020-01-10

**Authors:** Xia Xu, Hong Chen, Qi Zhang, Fei Lyu, Yuting Ding, Xuxia Zhou

**Affiliations:** College of Food Science and Technology, Zhejiang University of Technology, Hangzhou 310014, China; xuxia@zjut.edu.cn (X.X.); 18868195616@139.com (H.C.); zhangqi0309@Outlook.com (Q.Z.); lvfei_zju@163.com (F.L.)

**Keywords:** oil droplet size, interfacial protein film, myofibrillar proteins, dynamic rheological properties, interfacial shear rheology

## Abstract

The effects of oil droplet size and the formation of an interfacial protein film (IPF) on silver carp myofibrillar protein (MP)–oil composite gels were studied. MP- or Tween 80-stabilized camellia seed oil emulsions with different droplet sizes were prepared and added to MPs to prepare composite gels. The oil droplet size of the Tween 80-stabilized emulsion was significantly smaller (*p* < 0.05) than that of the MP-stabilized emulsion with the same homogenization speed. However, polymerization of Tween 80-stabilized emulsions during the preparation of the composite gels was found. Composite gels with the MP-stabilized emulsions of a small droplet size showed significantly improved water-holding capacity, texture, and dynamic rheological properties. Interfacial shear rheology studies revealed that the storage modulus (G’) of the MP-stabilized emulsion composite gels was higher than that of the Tween 80-stabilized gels, and the tan δ of the MP-stabilized oil emulsion composite gels was smaller than that of the Tween 80-stabilized gels, indicating that stronger elastic gel structures were formed. These results suggested that the IPF formed in the MP-stabilized emulsion helped stabilize the oil droplets embedded in the protein gel network, and the smaller the droplet size, the more stable the composite gel. This work provides a better understanding of how oil emulsions interact with protein and affect the properties of MP–oil composite gels.

## 1. Introduction

Surimi is a high-protein seafood product made from processed fish. During the production of surimi, fat and sarcoplasmic components of minced fish are usually washed away to obtain a crude concentrate of myofibrillar proteins (MPs) that has good gelling capacity [1]. It is relatively tasteless and odorless and is used especially to produce imitation crab meat and lobster meat. Functional food products supplemented with ingredients with specific health benefits have gained increasing attention, and increasingly more research has focused on improving the nutritional and functional qualities of surimi-based products with the addition of exogenous lipids [2].

Apart from the nutritional strengthening effects, lipids have been reported to have a great influence on the texture and sensory and flavor characteristics of surimi products [3,4]. There are many studies on the effects of lipid types as well as addition and substitution (high-quality oils instead of animal fat) on food quality [5,6,7]. However, little information is available on the mechanism of how oils affect the textural and functional properties of surimi protein gel to date. Studies on the interactions between surimi protein and exogenously added oil might be able to provide clarification of the underlying mechanism.

It is already known that during the gelation of surimi protein, the added oils are mixed into small fat droplets and fill the network void of the myofibrillar protein gel matrix in the form of fillers or copolymers in the protein gel and, at the same time, are stabilized by the three-dimensional network structure, thereby reducing gel porosity and reinforcing the gel structure [4,8]. Therefore, for better emulsification effects, lipids are usually added to surimi in the form of pre-emulsified oil emulsions. Additionally, the emulsifying properties of the oil emulsion, such as the oil droplet size, were reported to affect the rheological behavior and structural properties of the protein–oil composite gel. Sala et al. found that the fracture strain of gels incorporated with nonaggregated, bound droplets decreased with the decreasing size of the oil droplet; in addition, gelatin gels with whey protein isolate (WPI) and unbound droplets were less elastic and more viscous than those without added WPI [9]. Wu et al. found that reducing the oil droplet size induced an increase in the penetration force of the composite gels of an olive oil emulsion and myofibrillar proteins (*p* < 0.05) [10]. Decreasing the oil droplet size resulted in an increase in the droplet number and much smaller oil droplets that have larger total surfaces, which enabled them to interact with the gel network more extensively [3,4]. Gani and Benjakul reported that compared with the direct addition of virgin coconut oil, the incorporation of a virgin coconut oil nanoemulsion significantly (*p* < 0.05) increased the likeness and whiteness of surimi gel but caused no deterioration of the textural properties [5].

Types of emulsifier and gelation methods also affect the physical and chemical properties of protein and oil composite gels. Studies by Ben-Harb et al. indicated that fat strengthened the mechanical properties of pea gels and, compared to that of gels prepared with thermal and acid treatments, gels induced by chymosin and transglutaminase had a higher strain at rupture [11]. Abhyankar et al. emulsified sunflower oil with both whey protein and sodium caseinate and found that the type of the emulsifier protein influenced the rheological behavior of whey protein gels with added emulsified oil droplets [12]. These differences might partly be related to the stability of the emulsification system. Gordon and Barbut reported that the dispersed fat globules and the hydrophobic regions of MPs will interact with each other to form an interfacial protein film (IPF) during the formation of a fat and MP gel network, which could act as a filler and stabilize oils that have been separated into small globules in the composite gels [8]. Therefore, the IPF might play a vital role in the formation and stability of oil-protein gel systems.

In previous studies, we studied the effects of lard and camellia seed oil with different concentrations on the physical, chemical, and microstructural properties of fish myofibrillar protein-oil composite gels and found that the existence of an external oil significantly increased the gel strength, water-holding capacity (WHC), whiteness, storage modulus (G’), and loss modulus (G’’) of surimi myofibrillar gels [13]. However, little is known about the underlying mechanisms. Therefore, in the present study, we aimed to elucidate how camellia seed oil emulsions affect the properties of MP–oil composite gels, focusing on oil droplet size and IPF formation analysis, and for the preparation of oil emulsions, both MPs and Tween 80 were used as emulsifiers. Proteins are effective emulsifiers that are beneficial to the formation and stability of oil-in-water emulsions. Tween 80 is a nonprotein emulsifier and a nonionic surfactant, and fat droplets stabilized by Tween 80 are not expected to be bound to the protein in the gel matrix [14,15]. The formation of the IPF in the emulsions was imaged by confocal laser scanning microscopy (CLSM), and the breaking force, deformation, WHC, rheological properties, microstructure, and interfacial shear rheology of the composite gels were analyzed to clarify the effect of the interaction between proteins and oil droplets in a composite gel system.

## 2. Results and Discussion

### 2.1. Droplet Size and Distribution of Emulsions

The dynamic light scattering (DLS) method is useful for determining the droplet size and its distribution in emulsions with sufficient scattering contrast [16]. Table 1 shows the size and polydispersity index (PDI) of the emulsion droplets homogenized at different speeds. The size of the MP-stabilized emulsion droplets decreased significantly (*p* < 0.05) from 683.60 nm to 265.61 nm when the homogenization speed increased from 5000 to 15,000 rpm. The size of the droplets in the Tween 80-stabilized emulsions first decreased from approximately 165.02 nm to 15.79 nm (*p* < 0.05) and then remained constant at approximately 12.36 nm, which was significantly smaller than that of the MP-stabilized emulsions at the same homogenization speed (*p* < 0.05). The interfacial tension decreasing abilities of emulsifiers and the interfacial adsorption rate might explain the differences in droplet size. Compared with high molecular weight proteins, Tween 80 has a low molecular weight and can spread to the oil–water interface faster and reduce the interfacial tension to a greater extent [17,18].

In addition to droplet size, differences in the PDI of oil emulsions were observed. The PDI of the MP-stabilized emulsions decreased significantly (*p* < 0.05) with increasing homogenization speed, while that of the Tween 80-stabilized emulsions remained constant. At the same homogenization speed, Tween 80-stabilized emulsions had a significantly (*p* < 0.05) narrower droplet size distribution than that of the MP-stabilized emulsions, as indicated by the PDI results. As a small-molecule emulsifier, Tween 80 might be more active than a protein emulsifier, and it tends to form a compact flow adsorption layer at the interface but not a viscoelastic layer on the surface of the fat globule, while protein emulsions tend to flocculate, resulting in the formation of a tighter oil droplet floc, enhancing the droplet dispersion and widening the distribution width [19,20].

### 2.2. Microstructures of the Composite Gels

**Figure 1:**
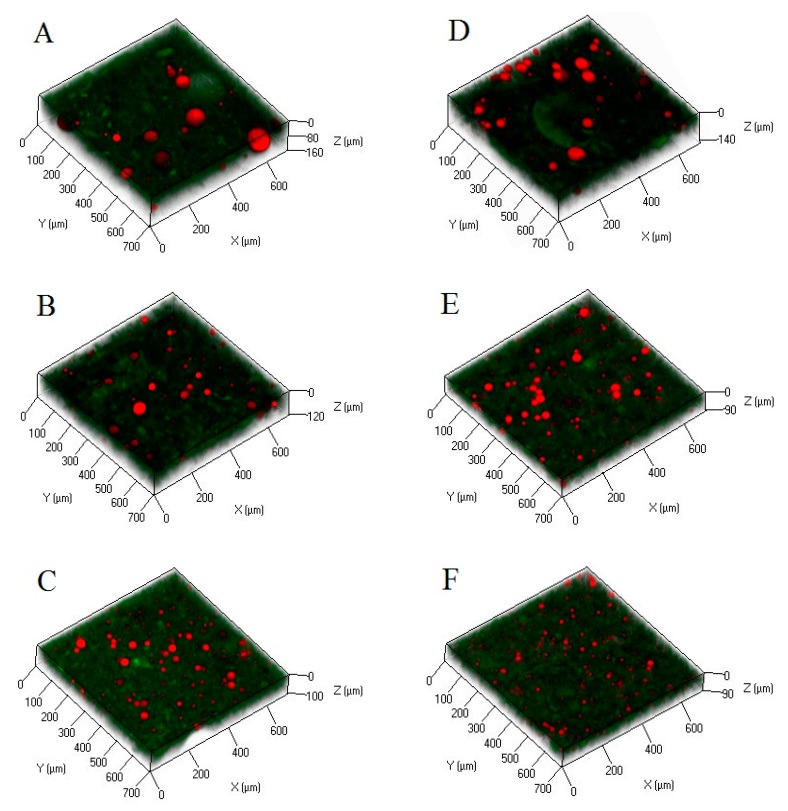
Microstructure of gels containing MP- or Tween 80-stabilized camellia seed oil emulsions. (**A**–**C**) = MP-stabilized emulsions homogenized at a speed of 5000 rpm, 10,000 rpm, and 15,000 rpm, respectively; (**D**–**F**) = Tween 80-stabilized emulsions homogenized at a speed of 5000 rpm, 10,000 rpm and 15,000 rpm, respectively. Fats are shown in red, and proteins are shown in green.

The microstructure images of the composite gels with added oils emulsified with different homogenization speeds using MPs (A–C) or Tween 80 (D–F) as the emulsifier are shown in Figure 1. Although DLS analysis showed that the oil droplets of the Tween 80-stabilized emulsion were significantly (*p* < 0.05) smaller than those of the MP-stabilized emulsion in the initial stage of the emulsification process, oil droplets in the composite gels were close in size. This indicated that although the protein emulsifier cannot effectively reduce the interfacial tension of the emulsion, its oil emulsion has higher stability in the composite gel than that of Tween 80. Protein molecules can form a gel-like interface film through noncovalent interactions to wrap oil droplets, regardless of whether the interface was a saturated monolayer or multilayer adsorption [17,18]. The conformational changes of the interface proteins will promote the formation of protein polymerization through thiol disulfide interchange reactions [3]. Considering that the adsorption of proteins at the interface is irreversible, these interactions can form a highly viscoelastic interface film to prevent coalescence [19,20]. Therefore, although the particle size, oil droplet distribution, and dispersibility of the Tween 80-stabilized emulsion droplets were smaller than those of the MP-stabilized ones in the stage of emulsion formation, they are prone to aggregate rapidly during the preparation of the gel. It can also be clearly observed that the Tween 80-stabilized emulsion droplets polymerized and bound with other oil droplets in the gels (Figure 1D,E), while the MP-stabilized emulsion droplets were relatively independent of each other (Figure 1A–C). These results suggest that the formation of IPF in MP-stabilized emulsion droplets helps stabilize the oil droplets that are regularly embedded in the protein gel network.

### 2.3. Effect of Oil Droplet Size and IPF on Textural Characteristics of Gels

**Figure 2:**
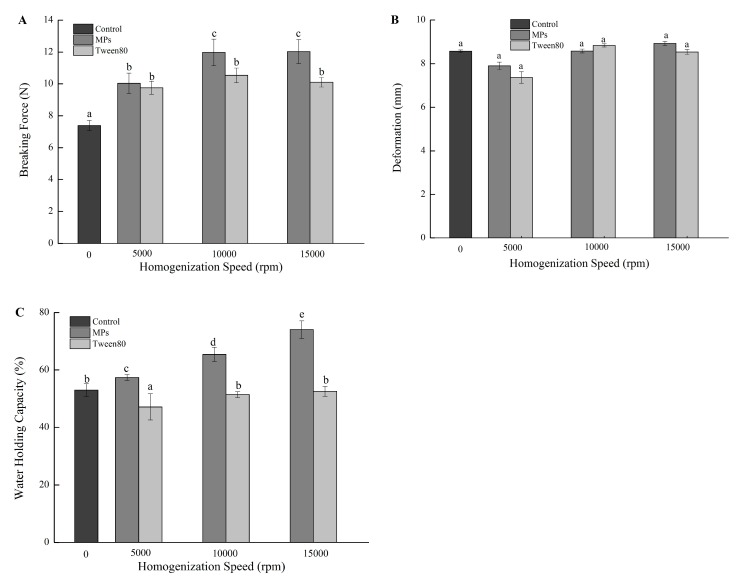
Breaking force (**A**), deformation (**B**), and the water-holding capacity (**C**) of gels containing MP- or Tween 80-stabilized camellia seed oil emulsions. Bars represent the standard deviation (*n* = 3). Different letters above bars indicate significant differences (*p* < 0.05).

The textural characteristics of the control MP gel and the composite gels are shown in Figure 2A,B. The breaking force of the composite gels was significantly higher than that of the control (*p* < 0.05), while the deformation of the composite gels was not significant different (*p* > 0.05). With the decrease of the oil droplet size in the MP-stabilized emulsion, the breaking force of the composite gel first increased (*p* < 0.05), reaching a maximum at a homogenization speed of 10,000 rpm and then remained constant. However, no significant differences were found for the composite gels made with the Tween 80-stabilized emulsions. Fat can be scattered into small globules, which can interact with salt-soluble MP membranes through disulfide linkages and hydrophobic interactions to form stable structures during the emulsification process. Zhuang et al. indicated that emulsified fat globules could result in a more compact structure through occupying void spaces in the composite gel networks [21]. Moreover, compared with larger oil droplets, smaller oil droplets that have a greater total surface area can usually interact with the gel network more extensively [3,4].

Compared with those made with Tween 80-stabilized oil emulsions, the breaking force of gels with the MP-stabilized emulsions was generally higher, and significant differences (*p* < 0.05) were found for the gels made with oil emulsions of smaller droplet size. Depending on the effects of oil droplets on the gelling properties, particles can be classified as inactive or active [22]. Usually, inactive fillers hardly combine with molecules in the gel matrix, while active fillers can interact with these molecules with strong chemical forces, which results in increased gel strength [15]. Wiedenmann et al. found an integration of solid oil nanoparticles (SLNs) stabilized by β-lactoglobulin in the gel network, while SLNs emulsified with Tween 20 did not participate in the formation of the gel network [23].

### 2.4. Effect of Oil Droplet Size and IPF on the WHC of Gels

WHC is one of the representative indicators for evaluating the quality of protein gels. Compared with that of the MP gel, the WHC of the composite gels with MP-stabilized oil emulsions significantly increased (*p* < 0.05), and the smaller the emulsion droplet, the greater the WHC (*p* < 0.05, Figure 2C). IPF can act as a physical barrier to the migration of emulsified fat globules and allow for the protein gel network matrix to be further agglutinated, limiting the flow of water and lipids through physical action [13]. In addition, smaller oil droplets have larger effective surface areas that can come into contact with protein and induce stronger interactions between the oil and the MPs to form a denser gel network structure [3,4]. Zhao et al. prepared a soybean oil pre-emulsion with a reduced particle size by ultrasound treatment and found that the emulsion can effectively improve the textural characteristics and lipid- and water-binding capacities of MP sol gels [2].

However, the addition of Tween 80-stabilized oil emulsions homogenized at 5000 rpm significantly decreased the WHC of the composite gels (*p* < 0.05). This is consistent with the results reported by Chen and Dickinson, who found that the rheological properties of the emulsion-filled gel could be improved with oil droplets stabilized by whey protein, while those stabilized by Tween 20 weakened the gel network [22]. Chojnicka et al. also found that the friction coefficient of WPI gels with Tween 20-stabilized emulsion oil droplets was lower than that without oil [24]. Presumably, the oil droplets stabilized by Tween 20, which had inactive interfacial membranes, were not embedded into the matrix in the gelatin gels. Therefore, Tween 80-stabilized emulsion oil droplets cannot be stably filled in the protein gel network structure, and excessive oil droplets formed by aggregation may even destroy the gel network structure, leading to increased dehydration and reduced WHC.

### 2.5. Effect of Oil Droplet Size and IPF on the Dynamic Rheological Behavior of Gels

**Figure 3:**
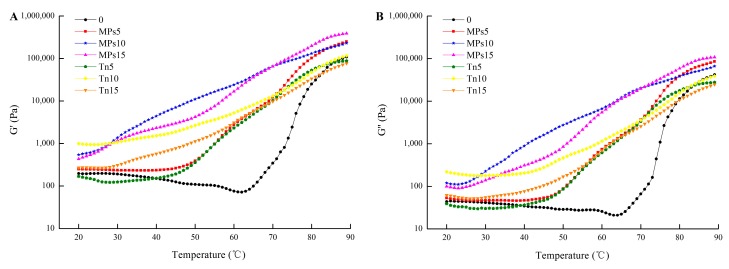
Storage modulus (G′) (**A**) and loss modulus (G″) (**B**) during heating of gels containing MP- or Tween 80-stabilized camellia seed oil emulsions. 0 = control; MPs5, MPs10, and MPs15 = gels with MP-stabilized emulsions homogenized at 5000, 10,000, and 15,000 rpm, respectively; Tn5, Tn10, and Tn15 = gels with Tween 80-stabilized emulsions homogenized at 5000, 10,000, and 15,000 rpm, respectively.

The G’ and G’’ values of the composite gel increased with the addition of oil emulsions (Figure 3). Generally, these values for gels made with MP-stabilized emulsions were higher than those made with Tween 80-stabilized emulsions, especially the G’ of the gels homogenized with Tween 80-stabilized emulsions at 5000 rpm, which was even lower than that of the control with the increasing temperature from 20 to 40 °C. The gels with lower homogenization speed mainly shift the “gel point” to lower temperature. It is probable the larger oil droplets destructed the protein structure and further reduced the gel stability, and then the gel point shifted to a lower temperature. Previous studies indicated that increased protein aggregation in MP gels was induced by the addition of oils by increasing the β-sheet content and decreasing the α-helix content [25]. Greater aggregation might be related to higher G’ and a more ordered protein network structure [26]. Moreover, the oil droplet surface component combined with the continuous protein matrix may be an important factor that determines the influence of oil droplets on the rheological behavior of the composite gel [15], and incorporation of unbound oil droplets will decrease the gel modulus [27]. Kirimlidou et al. found that oil droplets dispersed adequately within a continuous gelatin matrix could function as active fillers, exert a significantly increased modulus of the composite gelatin gel and also affect the viscoelastic parameters of the gel, suggesting that the oil droplets were incorporated within the composite gel matrix [27].

**Figure 4:**
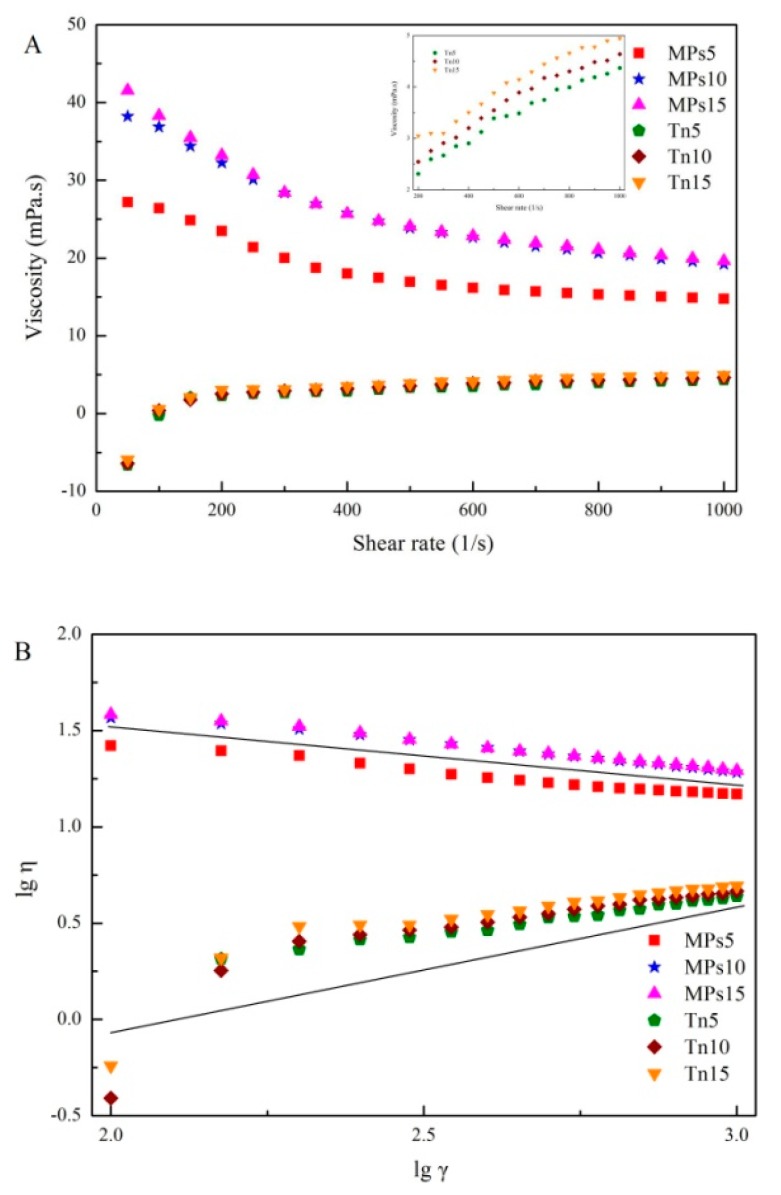
(**A**) Viscosity of MP- or Tween 80-stabilized camellia seed oil emulsions at different homogenization speeds. (**B**) Dependence of lgη versus lgγ. MPs5, MPs10, and MPs15 = gels with MP-stabilized emulsions homogenized at 5000, 10,000, and 15,000 rpm, respectively; Tn5, Tn10, and Tn15 = gels with Tween 80-stabilized emulsions homogenized at 5000, 10,000, and 15,000 rpm, respectively.

Figure 4A shows the viscosity of the emulsions as a function of the shear rates. The viscosity of the MP-stabilized emulsions generally decreased with increasing shear rate from 0 to 1000 s^−1^, and the smaller the droplet size, the faster the decrease of the viscosity. The viscosity of the Tween 80-stabilized emulsions generally increased with increasing shear rate regardless of the droplet size. The changes in the viscosity tended to be stable for the MP-stabilized emulsions from a shear rate of 600 s^−1^ onward and for Tween 80-stabilized emulsions from a shear rate of 200 s^−1^ onward. In other words, the fluids exhibit Newtonian fluid behavior when the shear rate reaches 600 s^−1^ or 200 s^−1^ for the MP-stabilized and the Tween 80-stabilized emulsions, respectively [2,28,29].

To better understand the solution behavior of the emulsions, the shear viscosity curves were fitted with the Oswald-DE Waele power-law equation η = Kγn^−1^, where η is the shear viscosity, K is a consistency factor that is proportional to the apparent viscosity, γ is the shear rate, and *n* is the flow index [30]. The fluid can be considered a pseudoplastic fluid if the *n* value is between 0 and 1 [31]. The values of K and *n* are shown in Table 1. The consistency factor K of the MP-stabilized emulsions increased significantly (*p* < 0.05) with the decrease of oil droplet size, and the consistency factor K of the MP-stabilized emulsions increased significantly (*p* < 0.05), while that of the Tween 80-stabilized emulsions did not change obviously. The K values of the MP-stabilized emulsions were significantly higher (*p* < 0.05) than those of the Tween 80-stabilized emulsions, indicating that the former was more stable. There was no significant change in the flow index *n* of the MP-stabilized emulsions, while that of the Tween 80-stabilized emulsions changed significantly with the decrease of oil droplet size (*p* < 0.05). In addition, because the droplet size of the Tween 80-stabilized emulsion was smaller and most of the oil interface was covered by Tween 80, a stronger interaction between oil droplets was allowed, which resulted in a stronger resistance to flow and a higher *n* value for the Tween 80-stabilized emulsion [32]. These results indicated that the MP-stabilized emulsions with smaller oil droplet sizes were pseudoplastic fluids with relatively strong shear-thinning abilities [2,29,31].

**Figure 5:**
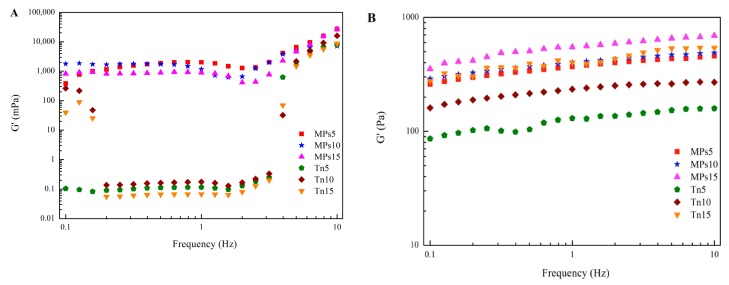
Storage modulus (G′) of MP- or Tween 80-stabilized camellia seed oil emulsions (**A**) and composite gels (**B**) across different frequencies at 25 °C. MPs5, MPs10, and MPs15 = gels with MP-stabilized emulsions homogenized at 5000, 10,000, and 15,000 rpm, respectively; Tn5, Tn10, and Tn15 = gels with Tween 80-stabilized emulsions homogenized at 5000, 10,000, and 15,000 rpm, respectively.

The changes in G′ and G″ of the emulsions with increasing frequency at 25 °C are illustrated in Figure S1. G′ and G″ remained constant, and G′ was lower than G″ in the low frequency area. In the high-frequency region, the curves of G′ and G″ first decreased and then increased quickly, and the values became closer, especially for the smallest MP-stabilized emulsion oil droplet, where G′ and G″ were basically equivalent, indicating that emulsions tended to form a weak gel structure in the high-frequency region and that a smaller oil droplet emulsion better formed a weak gel structure. Moreover, G′ increased with decreasing oil droplet size, and the G′ of the MP-stabilized emulsion was higher than that of the Tween 80-stabilized emulsion (Figure 5A). Gomes et al. found that colloidal interactions in an emulsion are important for the rheological properties of suspensions [32]. Compared with the Tween 80 emulsion, which allowed for a stronger interaction between oil droplets, the existence of protein molecules on the surface of the negatively charged droplets might increase the distance between droplets, suggesting the importance of an IPF.

### 2.6. Interfacial Shear Rheology of Gels

To maintain the frequency dependence (0.1–10 Hz) of G′ and G″ of the composite gels well within the linear viscoelastic region, a strain rate of 0.1% was implemented for the determination of G′ and G″ (Figure S2). The microstructures of the composite gels were analyzed by small-amplitude oscillation tests in the linear viscoelastic range [28]. For all of the composite gels, regardless of oil droplet size or emulsifier, an increase in G′ with increased frequency was observed, and G″ was always lower than G′ in the measured frequency range. The parameter tan δ, which is equal to G″/G′, is another index that can reflect the rheological behavior of gels; a conventional weak gel is typically indicated when tan δ > 0.1, and a traditional elastic gel is represent by tan δ < 0.1 [33]. The tan δ values of the composite gel made with MP-stabilized emulsions were smaller than those made with Tween 80-stabilized emulsions, illustrating that a stronger elastic gel structure was formed. The G′ of the composite gels with MP-stabilized emulsions was also higher than that with Tween-80 stabilized emulsions (Figure 5B). The increase in G′ might indicate that MP-stabilized emulsion droplets could act as active fillers in the protein gel matrix [22,34]. The entanglements increased with the existence of small oil droplets, and IPF might be beneficial for the formation of the composite gel network.

## 3. Materials and Methods

### 3.1. Materials

Live silver carp purchased from a local market were transported to the laboratory in a plastic bag with water. The live fishes were immediately killed by a physical blow to the head and then beheaded, gutted, and washed for use. Commercial camellia seed oil (CSO) was obtained from a local supermarket. All the reagents used were of analytical grade.

### 3.2. MP Preparation

MPs were prepared as described by Zhou et al. [13]. Ice-cold phosphate buffer and NaCl solutions were used during MP extraction to prevent protein denaturation and hydrolysis. The entire process was carried out at 4 °C. After pretreatment, the silver carp were minced and homogenized with 25 mM phosphate buffer (Na_2_HPO_4_/NaH_2_PO_4_, pH 6.8) for one minute and centrifuged (CR21GII, Hitachi, Japan). Then, the resuspended pellet was homogenized repeatedly and centrifuged. The obtained pellet was then homogenized in phosphate buffer, and the collected pellet was dissolved in a NaCl solution (0.1 M). The resulting pellet of MPs after homogenizing and centrifuging was stored at 4 °C before use.

### 3.3. Oil Emulsion Preparation

CSO (6 g) and 24 g of 1% diluted MPs (0.05 M Na_2_HPO_4_/ NaH_2_PO_4_, 0.6 M NaCl, pH 6.8) or Tween 80 solution were mixed. Next, the mixture was homogenized for 1 min at different speeds (5000, 10,000 and 15,000 rpm) (Ultra-Turrax T-25, Janke & Kunkel IKA Labortechnik, Staufen, Germany) for preparation of the oil emulsions.

### 3.4. Composite Gel Preparation

The MPs were pre-dissolved in sodium phosphate buffer (0.05 M Na_2_HPO_4_/NaH_2_PO_4_, 0.6 M NaCl, pH 6.8). The prepared MP- or Tween 80-stabilized CSO emulsions were added under stirring conditions. The two were fully blended to obtain MP–CSO composite gels, each having a final mass of 30 g. Final concentrations of the MPs and CSO were 6% and 2%, respectively, in both gels added with MP- or Tween 80- stabilized CSO emulsion. The control samples only contained 6% MPs. Subsequently, the prepared suspensions in a glass container were sealed and heated at 80 °C. After heating, the gels were immediately cooled down with ice water and then kept at 4 °C for 18 h.

### 3.5. Determination of Oil Droplet Size

DLS (Zetasizer Nano-ZS900, Malvern Instruments, Malvern, UK) was used to measure the droplet size and polydispersity index (PDI) of the oil emulsions. The results used for data processing and analysis were obtained as the average of three measurements.

### 3.6. Texture Analysis

The mechanical properties of gels that were equilibrated to room temperature were measured with a texture analyzer (TA.XT Plus, SMS, Surrey, UK). A metal probe (cylindrical plunger 5 mm in diameter) was vertically penetrated into the cut surface of gel samples at a constant depression speed of 0.5 mm/s. The instrument settings were as follows: pretest and posttest speed, 2.00 and 1.00 mm·s^−1^, respectively; trigger force, 5.0 g; and displacement, 10 mm. Both the deformation and the breaking force of the gels were recorded.

### 3.7. WHC Measurements

The gel WHC was determined using a centrifugation process as described by Amiri et al. [35]. The prepared composite gel (6 g) was centrifuged at 10,000 rpm for 10 min, and the supernatant was removed. Then, the WHC (%) of the composite gels was calculated as follows: WHC (%) = (m_2_ − m_0_)/(m_1_ − m_0_) × 100%, where m_0_ is the tube weight (g); and m_1_ and m_2_ are the weight of the tube containing gels before and after centrifugation, respectively (g).

### 3.8. Dynamic Rheological Measurements

The dynamic rheological behavior of the composite gels was analyzed through a controlled stress rheometer (MCR302, Anton Paar Ltd., Graz, Austria) with parallel plate geometry (25 mm diameter). A fixed gap between the plates was preset to 0.5 mm. Gels (2 mL) with or without oil emulsions were carefully and gently loaded into the geometry. Then, the gels were equilibrated for 5 min before each analysis. The storage modulus (G′) and loss modulus (G″) were measured at a fixed frequency of 0.1 Hz and a sinusoidal strain of 2.85% at varying temperatures from 20 to 90 °C with a linear heating rate (2 °C·min^−1^).

### 3.9. Interfacial Shear Rheological Measurements

The MCR302 Rheometer equipped with a stainless-steel plate-to-pate geometry (75 mm diameter, 1 mm gap) was applied to measure the interfacial shear rheological properties of emulsions and composited gels. The prepared emulsions (10 mL) or composite gels (10 g) were separately loaded into the geometry, followed by equilibration for 5 min, and the two plates were filled with liquid without bubbles. The emulsion viscosity was recorded as the shear rate increased from 0.01 to 1000 s^−1^. To determine the linear viscoelastic region, a strain sweep at 1 Hz was conducted. G′ and G″ were measured as a function of frequency from 0.1 to 10 Hz at a constant strain (0.1%) and 25 °C.

### 3.10. Confocal Laser Scanning Microscopy (CLSM)

Composited gels were stained with 0.2% (*w*/*w*) Fast Green and 0.2% (*w*/*w*) Nile Red to visualize the protein phase (green colored) and the fat phase (red colored), respectively. The final concentrations of Fast Green and Nile Red in the composited gels were both 0.001%. CLSM graphs were obtained using a confocal laser scanning microscope (TCS SP5, Leica Microsystems CMS GmbH, Wetzlar, Germany) in single photon mode equipped with an Ar/Kr visible light laser. A 63× water immersion Leica objective lens (PL APO) with a numerical aperture (NA) of 1.25 was used.

### 3.11. Statistical Analysis

All experiments were performed three or six times. The data were analyzed with SPSS version 13.0 (SPSS Inc., USA). Multiple comparisons were performed based on the Duncan method with a significance level of 95%, and the data were plotted with Origin 8.6 software (OriginLab Corp., Northampton, MA, USA).

## 4. Conclusions

Both the physicochemical properties of oil and the droplet size of the oil emulsion influence the interfacial properties between oil–oil and oil–protein, which can affect the stability of the emulsions and further affect the properties of oil–protein composite gels. The present research indicated that oil aggregation dynamics and interfacial protein membranes play important roles in the rheological behavior of composite gels. Smaller oil droplets coated with salt-soluble MP membranes and connected or bound to proteins can act as active fillers and significantly improve the WHC, strengthen the texture of the composite gels, and increase the dynamics and interfacial shear modulus G′ and G″. The addition of oil emulsions of a large droplet size surface, for example, the oil emulsified by Tween 80 at 5000 rpm, resulted in a decrease in the gel WHC and modulus, leading to a destruction of the gel structure. These results demonstrated the effect of oil droplet size and highlighted the importance of IPF in composite gels made with oil emulsions.

## Figures and Tables

**Table 1:** The droplet size (DS), polydispersity index (PDI), K, and *n* of the myofibrillar protein (MP)- or Tween 80-stabilized camellia seed oil emulsions.

Index	MPs5	MPs10	MPs15	Tn5	Tn10	Tn15
DS (nm)	683.60 ± 37.26 ^e^	474.50 ± 8.30 ^d^	265.61 ± 10.15 ^c^	165.02 ± 38.15 ^b^	15.79 ± 0.75 ^a^	12.36 ± 1.25 ^a^
PDI	17.14 ± 2.78 ^c^	5.31 ± 0.63 ^b^	0.29 ± 0.02 ^a^	1.46 ± 0.51 ^a^	0.07 ± 0.01 ^a^	0.17 ± 0.02 ^a^
K	95.06 ± 5.62 ^b^	167.9 ± 10.44 ^c^	173.3 ± 9.35 ^c^	0.25 ± 0.002 ^a^	0.33 ± 0.01 ^a^	0.28 ± 0.002 ^a^
*n*	0.73 ± 0.03 ^a^	0.70 ± 0.01 ^a^	0.71 ± 0.05 ^a^	1.50 ± 0.02 ^b^	1.62 ± 0.03 ^c^	1.68 ± 0.02 ^d^

Different Letters (^a–e^) in a row within the same form of gels, including the control, indicate significant differences (*p* < 0.05). MPs5, MPs10, and MPs15 = gels with MP-stabilized emulsions homogenized at 5000, 10,000, and 15,000 rpm, respectively; Tn5, Tn10, and Tn15 = gels with Tween 80-stabilized emulsions homogenized at 5000, 10,000, and 15,000 rpm, respectively; K = consistency factor that is proportional to the apparent viscosity.

## Supplementary Materials

The following are available online. Figure S1: Storage modulus (G′) and loss modulus (G″) of MP- or Tween 80-stabilized camellia seed oil emulsions across different frequencies at 25 °C. Figure S2: Storage modulus (G′), loss modulus (G″), and tan δ of gels containing MP- or Tween 80-stabilized camellia seed oil emulsions across different frequencies.
