# Supplementary material for: Effects of Oil Droplet Size and Interfacial Protein Film on the Properties of Fish Myofibrillar Protein–Oil Composite Gels
Source: Molecules. 2020 Jan 10;25(2):289. doi: 10.3390/molecules25020289 (PMC7024232; doi:10.3390/molecules25020289)
Supplement: Supplementary file 1 [file molecules-25-00289-s001.pdf]

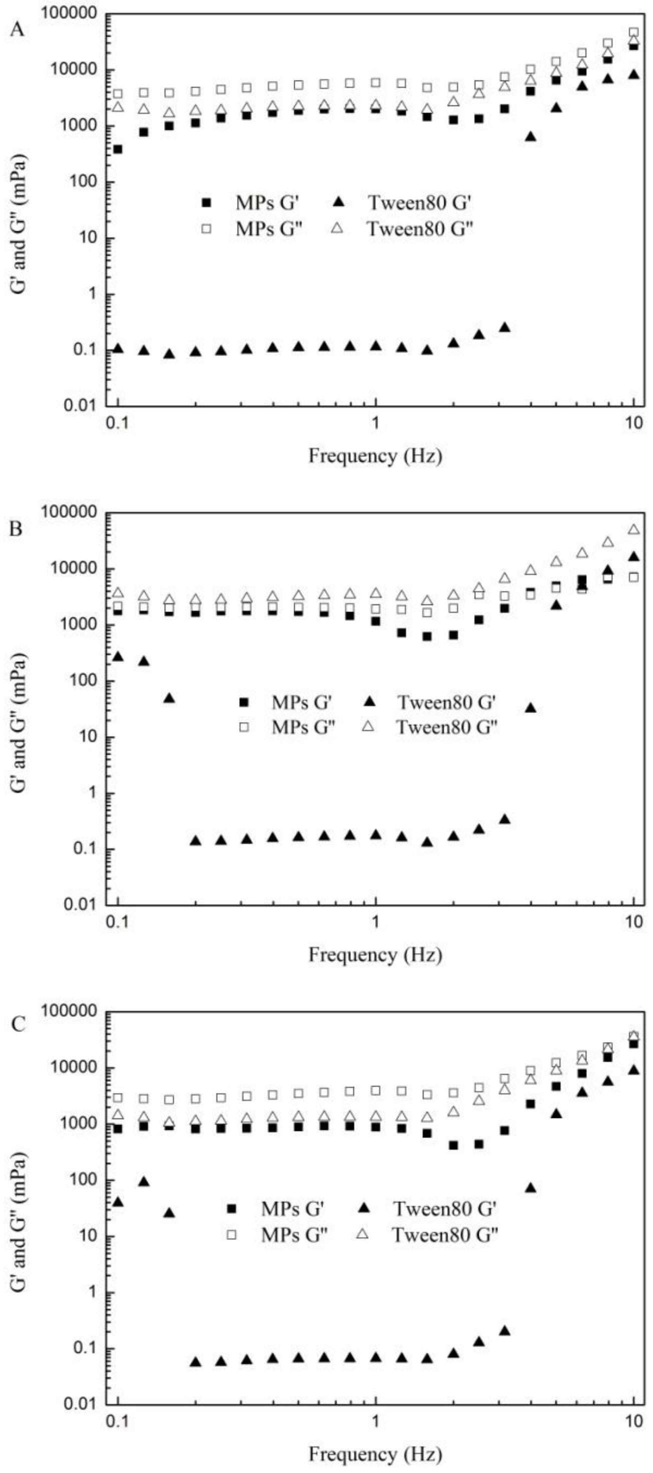

Figure S1. Storage modulus (G') and loss modulus (G'') of MP- or Tween 80-stabilized camellia seed oil emulsions across different frequencies at 25 °C. A, homogenized at a speed of 5000 rpm; B, homogenized at a speed of 10000 rpm; C, homogenized at a speed of 15000 rpm

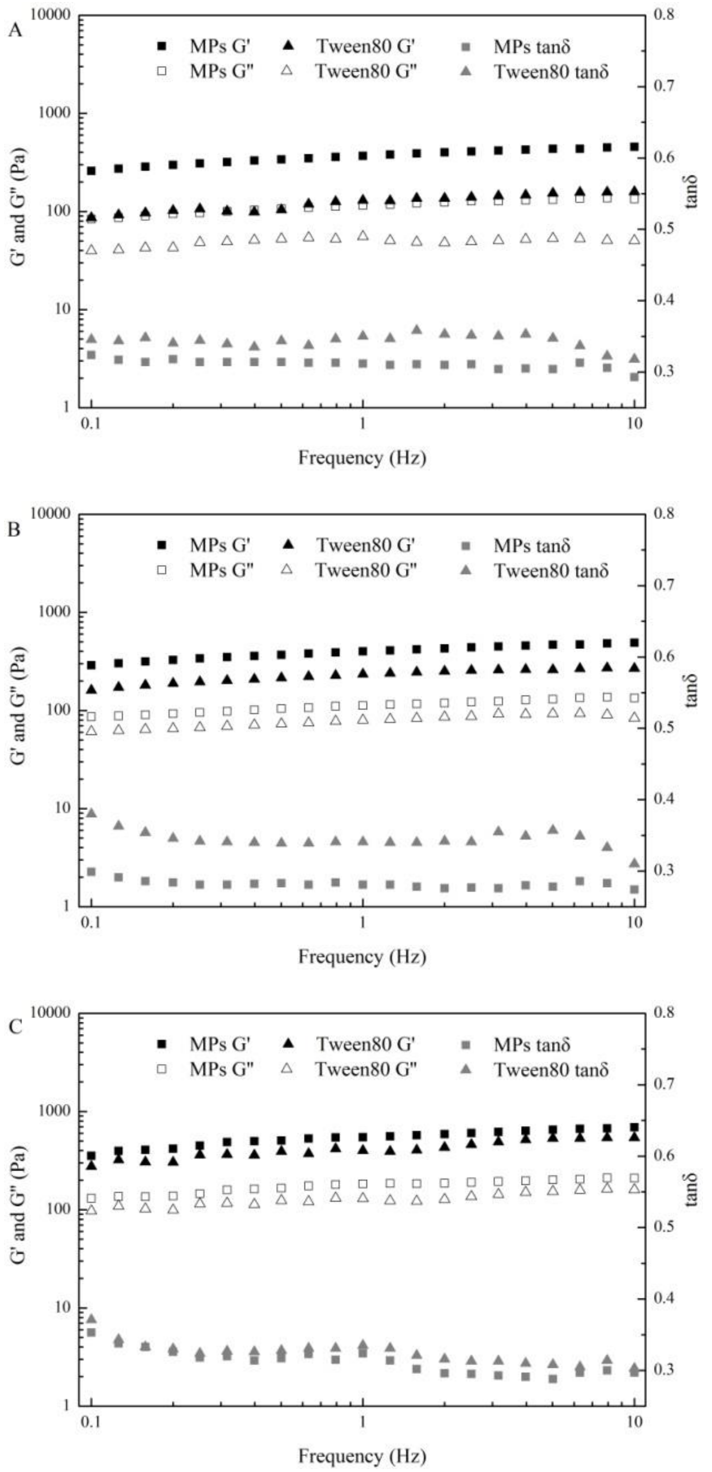

Figure S2. Storage modulus (G'), loss modulus (G'') and tan δ of gels containing MP- or Tween 80-stabilized camellia seed oil emulsions across different frequencies. A, homogenized at a speed of 5000 rpm; B, homogenized at a speed of 10000 rpm; C, homogenized at a speed of 15000 rpm.
